# Insect-related sporotrichosis oculoglandular syndrome: A case report

**DOI:** 10.1016/j.mmcr.2026.100765

**Published:** 2026-01-17

**Authors:** Kosol Kampitak, Thanat Kampitak, Naree Warnnissorn, Arvemas Watcharakorn, Worakit Kaewnopparat, Panarat Hematulin

**Affiliations:** a Department of Ophthalmology, Faculty of Medicine, Thammasat University, Pathumthani, Thailand; b Department of Pathology, Faculty of Medicine, Thammasat University, Pathumthani, Thailand; c Department of Radiology, Faculty of Medicine, Thammasat University, Pathumthani, Thailand; d Department of Microbiology, Thammasat University Hospital, Thammasat University, Pathumthani, Thailand

**Keywords:** Sporotrichosis, Oculoglandular syndrome, Insect, Case report

## Abstract

Sporotrichosis oculoglandular syndrome is an infectious disease caused by the thermodimorphic fungus *Sporothrix* species. Humans can become accidentally infected through vegetative trauma and contact with cats. However, there are no reported cases of this disease being transmitted by insects. In this report, we present a case of insect-related sporotrichosis oculoglandular syndrome.

## 1 Introduction

Oculoglandular syndrome is an infectious disease of the eye characterized by granulomatous conjunctivitis and the spread of infection to nearby lymph nodes, such as the preauricular, submandibular, and cervical lymph nodes. *Sporothrix* species is one of the common causative organisms [1,2]. This thermodimorphic fungus is found in soil and vegetative matter, and is transmitted to humans through direct traumatic inoculation, such as from vegetative trauma [3,4], as well as through increasingly reported cases related to contact with cats [2,[5], [6], [7], [8]].

However, there have been no documented instances of this disease being spread by insects. Here, we describe a case of oculoglandular sporotrichosis associated with insect exposure.

## 2 Case presentation

A 38-year-old Thai female office worker presented with progressive swelling of the right eyelid, cheek, and neck over the course of one month (day −30). She reported that 2 weeks before (day −44), her right eye was accidentally struck by something while she was lying on her bed, and after rubbing her eye to remove it, a tiny black gnat-like insect was observed.

There was no other reported history of trauma from vegetative materials or contact with cats or other animals. Moreover, there were no pets or other animals near her house. Additionally, she had no underlying disease and had no gardening activities.

She was referred to our hospital after one week of unresponsive treatment with intravenous vancomycin and ceftazidime for periorbital cellulitis.

Upon examination (day 0), edema of the right upper and lower eyelids, and a small abscess on the right upper eyelid were detected. Enlargement of the right preauricular and cervical lymph nodes was palpated; however, no wounds or ulcers were observed on the eyelids or nearby skin.

**Fig. 1:**
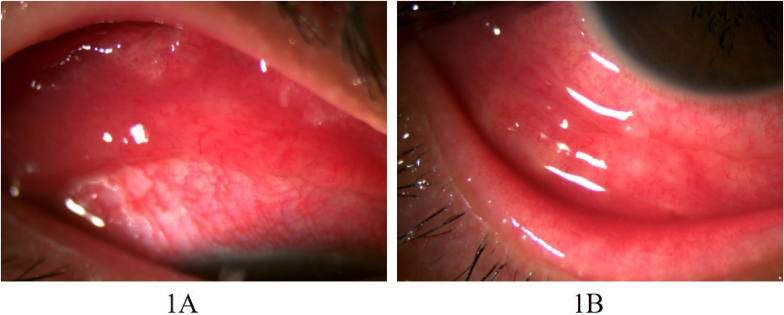
Multiple conjunctival nodules on the upper (1A) and lower (1B) conjunctiva of the right eye.

Multiple conjunctival nodules were identified on the upper and lower conjunctiva of the right eye (Fig. 1). The cornea was clear, and no cells were found in the anterior chamber or vitreous; the fundus examination showed normal findings.

**Fig. 2:**
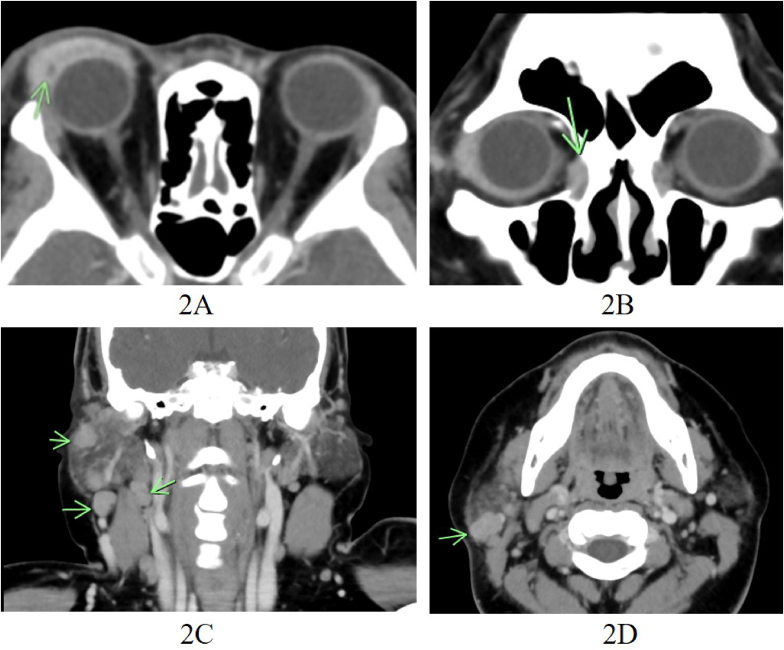
Contrast-enhanced computed tomography scan **2A.** An ill-defined enhancing soft tissue density lesion involving the right upper eyelid, with a rim-enhancing lesion inside, compatible with infection with an abscess **2B.** The ill-defined soft tissue enhancing lesion at anteromedial aspect of the right orbit extending to superior aspect of the right nasolacrimal duct, probable another infectious site **2C and 2D.** Multiple enlarged and subcentimeter lymph nodes at right cervical region (2C) and right intraparotid region (2D).

A contrast-enhanced computed tomography scan revealed enhancing soft tissue density lesions involving the right upper and lower eyelids, with a rim-enhancing lesion consistent with preseptal cellulitis and abscesses within the right upper eyelid, an ill-defined soft tissue enhancing lesion at anteromedial aspect of the right orbit extending to superior aspect of the right nasolacrimal duct, probably another infectious site, and a mild swelling of the right lacrimal gland suggestive of a secondary inflammatory change. Multiple enlarged and subcentimeter lymph nodes were noted in the right cervical group I–V and the right intraparotid region (Fig. 2).

Serum Bartonella IgG and IgM, Melioid titer, TPHA, and RPR yielded negative results. Conjunctival nodules were subsequently biopsied, and the eyelid abscess was drained.

Culture for aerobe, culture for anaerobe, PCR for TB, PCR for bacteria, and PCR for fungus from both conjunctival biopsy and pus were all negative.

**Fig. 3:**
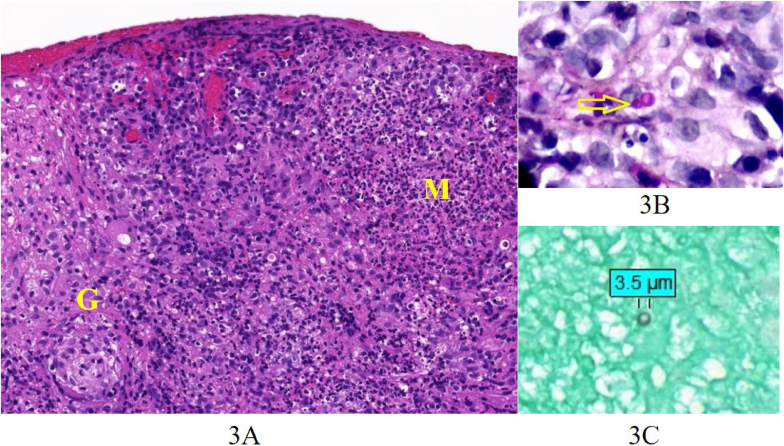
Conjunctival biopsy **3A.** Granuloma (G) and microabscess (M) (H&E, 200x) **3B.** Budding yeast (PAS, 400x) **3C.** Yeast (GMS, 400x).

Tissue biopsy revealed granulomas with microabscesses and rare budding yeasts with round to oval shape, measuring 2–5 μm in size (Fig. 3); negative for organism in Warthin-Starry stain, AFB, and Treponema immunohistochemistry.

**Fig. 4:**
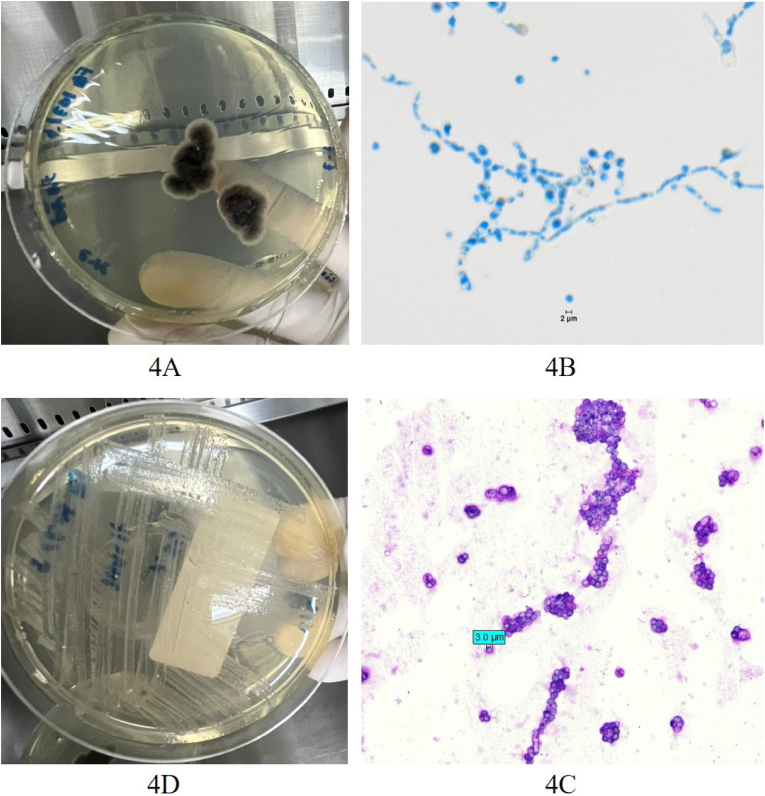
Fungal Culture (4A,B at 25 °C; 4C,D at 37 °C) **4A**. Mold colonies on Sabouraud Dextrose Agar after 7 days (surface of colony) **4B**. Hyaline septate hyphae with typical flower-like arrangement of conidial clusters (Lactophenol Cotton Blue stain) **4C.** Yeast colonies on Sabouraud Dextrose Agar after 2 days **4D.** Spherical and oval budding yeast cells (Wright stain).

Fungal culture identified a thermodimorphic fungus (mold at 25 °C; yeast at 37 °C), specifically *Sporothrix* species, based on phenotypic characteristics (Fig. 4). Thus, sporotrichosis oculoglandular syndrome was diagnosed.

The patient received oral itraconazole at a dosage of 400 mg daily. In the six months of treatment, she achieved complete resolution without adverse side effects.

## 3 Discussion

In cases presenting with eye inflammation and periorbital tissue swelling, misdiagnosis and mismanagement as periorbital cellulitis can occur [9,10]. However, if there is no improvement in the condition after treatment, it may warrant further investigation.

In the present case report, our patient was initially treated for periorbital cellulitis. But there was no response, leading to a referral to our hospital.

Detection of the spread of infection to adjacent lymph nodes serves as a clue for the diagnosis of oculoglandular syndrome. In a previous case report, enlargement of lymph nodes was not detected on initial examination, and the patient was treated for periorbital cellulitis. A computed tomography scan performed later revealed lymph node involvement, raising suspicion for oculoglandular syndrome and guiding appropriate treatment [10].

When diagnosing oculoglandular syndrome, the next step is to identify the causative organism. This syndrome can be caused by various organisms, including bacteria, fungi, or viruses. A biopsy of the infected lesions should be performed and sent for culture and pathological examination.

In the present case report, yeast cells were detected in the pathological specimen, and *Sporothrix* species was identified in the culture.

Sporotrichosis is an infectious disease caused by fungi of the genus *Sporothrix* [1,3,4]. This thermodimorphic fungus is found in soil, plants, and vegetative matter. The infection is transmitted to humans through direct traumatic inoculation from the matter contaminated with fungus, such as through vegetative trauma; hence the term “rose gardener's disease” is used [7].

Another mode of transmission, increasingly reported in recent decades, is through contact with cats [2,[5], [6], [7], [8]]. However, it is important to note that there is another etiological organism transmitted by cats which can also lead to oculoglandular syndrome. This organism is a bacterium named *Bartonella henselae*, which cause a disease commonly called “cat-scratch disease” [11].

In the present case report, there was no history of vegetative material trauma or contact with cats or other animals. The only evidence of eye trauma was that the right eye was struck by an insect before the onset of inflammation. Thus, this case may be reported as **“insect-related sporotrichosis oculoglandular syndrome”**.

Oculoglandular syndrome is a rare disease characterized by granulomatous inflammation of the eye and is associated with ipsilateral regional lymphadenopathy. The lesions can be classified into extraocular (eyelid, lacrimal system, and conjunctiva) and intraocular involvement (uveitis, endophthalmitis) [1].

In the present case report, all parts of the ocular adnexa (eyelid, lacrimal system, and conjunctiva) were involved, but there were no signs of intraocular involvement. Multiple conjunctival nodules were identified on the conjunctiva. A computed tomography scan revealed lesions involving the right upper and lower eyelids, the right nasolacrimal duct, the right lacrimal gland, the right cervical lymph nodes, and the right intraparotid region.

Itraconazole is the drug of choice for the treatment of sporotrichosis, typically for a duration of four to six months. Amphotericin B is reserved for systemic or disseminated cases [1].

Our patient achieved complete resolution following treatment with oral itraconazole at a dosage of 400 mg daily for six months.

Despite the absence of a history of vegetative material trauma or contact with cats, sporothrix-associated oculoglandular syndrome can be caused by insect contacts.

## CRediT authorship contribution statement

**Kosol Kampitak:** Conceptualization, Data curation, Investigation, Methodology, Supervision, Writing – original draft. **Thanat Kampitak:** Conceptualization, Data curation, Methodology, Project administration, Writing – original draft, Writing – review & editing. **Naree Warnnissorn:** Data curation, Investigation, Writing – review & editing. **Arvemas Watcharakorn:** Data curation, Investigation, Writing – review & editing. **Worakit Kaewnopparat:** Data curation, Investigation, Writing – review & editing. **Panarat Hematulin:** Data curation, Investigation, Writing – review & editing.

## Ethical form

Written informed consent was obtained from the patient or legal guardian(s) for publication of this case report and accompanying images. A copy of the written consent is available for review by the Editor-in-Chief of this journal on request.

## Funding source

There are none.

## Conflict of interest

There are none.
